# Predicting Physical Aggression among Schizophrenia Patients in Rural Communities of Southwestern China

**DOI:** 10.31083/AP46062

**Published:** 2025-08-26

**Authors:** Dongmei Wu, Tingting Liu, Quan Song, Changwei Li, Yuchuan Yue, Junlan Yang, Tao Li, Zixiang Ye

**Affiliations:** ^1^The Clinical Hospital of Chengdu Brain Science Institute, MOE Key Lab for Neuroinformation, University of Electronic Science and Technology of China, 610036 Chengdu, Sichuan, China; ^2^Florida State University College of Nursing, Vivian M. Duxbury Hall, Tallahassee, FL 32306-4310, USA; ^3^Pengzhou Fourth People’s Hospital (Pengzhou Mental Health Center), 611933 Chengdu, Sichuan, China; ^4^Department of Public Health, UT Southwestern Medical Center, Dallas, TX 75390, USA

**Keywords:** physical aggression, aggressions, chizophrenia, rural communities, prediction, China

## Abstract

**Objective:**

Physical aggression in schizophrenia patients carries significant societal implications. Previous studies on aggression prediction have primarily focused on hospitalized patients, overlooking specific rural community contexts in China. This study investigated multidimensional predictive factors to develop and validate a predictive model for predicting physical aggression in schizophrenia patients in rural communities in southwestern China.

**Methods:**

We used convenience sampling to select 889 confirmed patients with schizophrenia from 22 rural townships recorded by the Pengzhou Mental Health Center from September to November, 2019 for baseline survey. Sixty-two candidate factors were investigated using the Morningness-Eveningness Questionnaire, Multidimensional Fatigue Inventory, and Medication Coherence Rating Scale, and aggression was evaluated using the Modified Overt Aggression Scale during a 3-month follow-up. Logistic regression was used to construct a risk prediction model and the model was validated using the receiver operating characteristic (ROC) curve.

**Results:**

Two variable selection methods, least absolute shrinkage and selection operator and multivariate logistic regression, yielded two models: Model 1 with 27 variables and Model 2 with six variables. Both models demonstrated perfect discrimination, good calibration, and clinical utility. Model 2, with three historical and three modifiable factors, demonstrated greater utility for communities, which included physical aggression against others prior to the first episode of schizophrenia, the Modified Overt Aggression Scale score at first episode onset, mental fatigue, body mass index, experiences of discrimination, and aggression against objects before the first episode. Most predictors were non-specific to schizophrenia.

**Conclusion:**

These findings may help to alleviate the social discrimination and constraints faced by individuals with schizophrenia in rural communities, facilitating the provision of proactive mental health services. Furthermore, our results highlight body mass index, discrimination experiences, and mental fatigue as critical areas for rural community mental health nursing professionals.

**Clinical Trial Registration:**

No: ChiCTR1800015219. https://www.chictr.org.cn/showproj.html?proj=25941.

## Main Points

1. This study found that the aggressive behavior of rural schizophrenia patients may not be related to psychiatric symptoms.

2. This study found that physical attacks before onset of illness in rural patients are the most predictive of seizures after onset.

3. This study developed a 6-factor predictive model for effectively predicting aggressive behavior in rural schizophrenia, with body mass index (BMI), discrimination, and mental fatigue being crucial factors.

## 1. Introduction

In China, there are 6.331 million patients with severe mental disorders receiving regular medical follow-ups in the community, of which about three quarters have schizophrenia [1, 2]. According to a survey of 20,884 participants in agricultural areas of China, the weighted 1-month and lifetime prevalence rates of schizophrenia were 0.5% and 0.6% respectively [3]. Although the figure is lower than that in urban areas, based on the huge population in rural regions and the lack of mental health service resources, the medical treatment and community management of such a large group of schizophrenia patients have always been a difficult worldwide problem. With the development of society and the improvement of community residents’ mental health knowledge, the community’s understanding of schizophrenia has become more accurate and compassionate. The China 686 Project provides follow-up management, emergency response, free physical examinations, health education, and other services for patients with six types of severe mental disorders. Its long-term goal is to establish a patient-centered, function oriented, community-based, multidisciplinary mental health service system [4]. However, their views on the dangerousness, aggressiveness, and unpredictability of schizophrenic patients have remained stagnant [5]. Aggression is prevalent among schizophrenia patients, with rates varying across studies [6]. The rate of aggression in patients with schizophrenia increased from 30.7% in 2011 to 40.2% in 2015 in China [7]. The deep-rooted perception of the danger of schizophrenia in the community may constitute the primary source of stigmatization and discrimination. Notably, physical aggression against others, a concern for caregivers and community residents, has broader societal implications, including community instability, social isolation, and discrimination against mental health patients.

Numerous studies have extensively examined risk factors for aggression, leading to the development of various instruments to assess aggressive behaviors in individuals with psychosis. However, a systematic review of studies on Chinese psychiatric patients pointed out that there was almost no assessment instrument that could accurately predict their risk of violence and there was a call for developing more accurate prediction methods [8]. The establishment of predictive models could contribute to narrowing the gap between epidemiological evidence and individual practical assessments [9]. It is also beneficial for nurses to implement proactive control management of the risk of aggression in patients with schizophrenia, particularly aiding in enhancing the efficiency of nursing care for schizophrenia patients. Effective tools for evaluating aggressive behaviors in hospitalized patients have been proposed, leveraging their abundant and easily accessible clinical information [10]. However, there is evident heterogeneity in characteristics between community schizophrenic and clinical patients, such as demographic characteristics, psychiatric symptoms, treatment adherence, and physical and cultural environment factors, which may impact their aggressive behaviors.

This study focuses on physical aggression towards others, which often leads to more restrictions and social discrimination against schizophrenia patients in rural areas of southwestern China, resulting in feelings of shame, low self-identity, and a continuous decrease in self-esteem. It also affects social function and results in less social support for patients, which not only seriously affects their quality of life, but also causes poor treatment compliance, thereby affecting their overall rehabilitation and recovery outcomes. In addition, the physical aggression of individuals with schizophrenia towards others exacerbates the management challenges for nurses and exacerbates the contradiction between the shortage of psychiatric nursing human resources and the complex risk management of schizophrenia patients. By investigating multidimensional predictive factors, our goal is to develop and validate a concise predictive model for estimating the likelihood of physical attacks on others within the next 3 months. In addition, we used a column chart commonly used for medical risk prediction to visualize the model, providing a convenient and effective decision support tool. This method makes the model more suitable for rural areas with limited internet access, in order to provide a more reliable basis for the management and treatment of schizophrenia in rural areas of Southwest China.

## 2. Materials and Methods

### 2.1 Study Design and Participants

This study includes 62 variable indicators, and according to the sample size calculation method in relevant research, the sample size should be 5–10 times that of the number of variables [11]. Based on the actual situation of schizophrenia cases in rural areas of southwestern China and considering a 20% inefficiency rate, the required sample size is at least 744 cases.

This research data originates from a longitudinal study, which we have named the Southwest China Rural Schizophrenia Longitudinal Study (SCRSLS). In this study, the baseline survey was conducted between September and November, 2019. Using a convenience sampling method, 22 rural townships in Pengzhou City’s Mental Health Center were selected to record confirmed patients with schizophrenia who participated in the China 686 Project. The baseline survey gathered data on all 62 candidate factors, including sociodemographic characteristics, disease-related attributes, and other pertinent variables. The information collected during the first 2 months served as the training dataset, while data gathered in the subsequent month constituted the testing dataset. A follow-up survey was carried out 3 months after the baseline study, spanning from December, 2019 to February, 2020. During this phase, information regarding participants’ engagement in physical aggression towards others over the past 3 months was recorded. The SCRSLS commenced in September 2019 with quarterly (every three months) surveys conducted among the participants. Data collection was paused between February and August 2020 due to the global outbreak of Coronavirus Disease 2019 (COVID-19) but resumed in September 2020 with continued quarterly follow-ups until December 2021, resulting in a total of seven follow-up surveys. While the SCRSLS involved repeated assessments of participants over time, the content of the follow-up surveys was adapted to capture evolving clinical and social factors relevant to schizophrenia management in rural settings.

The inclusion criteria comprised individuals who: (a) had been diagnosed with schizophrenia according to the Diagnostic and Statistical Manual of Mental Disorders (fifth edition); (b) were aged between 18 and 60 years; (c) demonstrated the ability to read, write, and speak Chinese; and (d) provided consent to participate, and whose legal guardians were informed about the study and provided consent to participate. Exclusion criteria included: (a) comorbidity with other mental disorders; (b) experiencing health conditions that impeded investigation tolerance, such as nervous system diseases, brain development disorders, severe trauma, and physical illnesses; and (c) involvement in other concurrent studies.

### 2.2 Procedure and Data Collection

All participants were individuals diagnosed with schizophrenia and were receiving community-based management. Self-rating and other types of reporting were conducted according to the instructions provided in the questionnaire. A total of 18 psychiatric nurses were trained and subsequently employed as investigators to conduct field surveys. During the field survey, investigators clarified any ambiguities in real time. Objective and practical elements included in the questionnaire, such as years of education, birthplace, employment, marital status, and similar details, were meticulously verified by the participant’s legal guardian on an item-by-item basis. In cases of discrepancy between the participant’s responses and those of the legal guardian, the response provided by the legal guardian was acknowledged and documented.

#### 2.2.1 Candidate Predictors

Table 1 (Ref. [12, 13, 14, 15, 16, 17, 18, 19, 20, 21, 22, 23, 24, 25]) displays the 62 candidate predictors investigated in this study, encompassing demographic characteristics (such as age, gender, and years of education), circadian rhythm [12] and substance use, smoking, alcohol consumption, physical conditions (body mass index, fatigue status [13, 14], etc.), characteristics and treatment of mental disorders (duration of schizophrenia, medication adherence [15, 16], insight and treatment attitudes [17], severity of psychotic symptoms [18], etc.), family and community-related information (parental schizophrenia, household monthly income, experiences of discrimination [19], life events [20], social performance [21], etc.), relevant experiences in childhood and adolescence (parental relationships, childhood trauma [22, 23], etc.), and history of aggressive behavior [24, 25]. Data were collected through tailored questionnaires and corresponding scales, detailed in Table 1.

**Table 1.** **Candidate predictors of physical aggression against others and related questionnaires used**.

Characteristics (Number of predictors)	Candidate Predictors	Scales Used
Demographic characteristics (13)	Age ^a^, gender, years of education ^a^, birthplace, main living place in the past 5 years, living alone, number of children ^a^, employment, marital status, satisfaction with marital status ^b^, having one or more sexual partners, sexually satisfied ^b^, satisfaction with entertainment activities in the past 3 months ^b^.	_
Circadian rhythm and substance use, smoking, alcohol consumption (4)	Circadian rhythm ^a,d^, smoking, alcohol abuse, substance abuse.	Morningness-Eveningness Questionnaire [12]
Physical conditions (8)	Body mass index ^a^, physical diseases, self-care ability in the past 3 months, chronic pain in the past 3 months, fatigue status (physical fatigue, reduced activity, reduced motivation, and mental fatigue) ^a,d^.	Multidimensional Fatigue Inventory [13, 14]
Characteristics and treatment of mental disorders (13)	First episode schizophrenia, schizophrenia duration (monthly) ^a^, number of antipsychotic medications taken in the past 3 months ^a^, psychiatric outpatient/inpatient service use in the past 3 months, medication adherence (behavior and attitude) ^a,d^, insight and treatment attitudes ^a,d^, severity of psychotic symptoms (anxiety, withdrawal, psychosis, activation, and hostility) ^a,d^.	Medication Adherence Rating Scale [15, 16]
		Insight and Treatment Attitudes Questionnaire [17]
		Brief Psychiatric Rating Scale [18]
Family and community related information (8)	Father/mother with schizophrenia, household monthly income (≤689.59 USD and >689.59 USD), perceived family economic status ^b^, perceived social status ^b^, experiences of discrimination ^a,d^, life events ^a,d^, social performance ^a,d^.	Modified Consumer Experiences of Stigma Questionnaire [19]*
		List of Threatening Events Questionnaire [20]
		Personal and Social Performance Scale [21]
Relevant experiences in childhood and adolescence (11)	Living with parents before the age of 16 years, parental relationship before the patient was 16 years old, father/mother smoked before the patient was 16 years old, father/mother drank alcohol before the patient was 16 years old, childhood trauma (emotional abuse, physical abuse, sex abuse, emotional neglect, and physical neglect) ^a,d^.	Childhood Trauma Questionnaire [22, 23]
History of aggressive behavior (5)	Aggressive behaviors existed before the first episode of schizophrenia (verbal aggression, aggression against objects, aggression against self, and aggression against others), aggressive behaviors at the onset of the first episode of schizophrenia ^a,d^.	Modified Overt Aggression Scale [24, 25]

Note. No superscript indicates binary variables (yes or no). Unspecified refers to using self-made questionnaires. Body mass index is calculated as weight in kilograms divided by height in meters squared. ^a^ Continuous variable. ^b^ Using a 5-point Likert type scale (from 1 = extremely dissatisfied to 5 = extremely satisfied). ^d^ Measuring with Scale. * Five discrimination experience items suitable for Chinese patients were selected from the Modified Consumer Experiences of Stigma Questionnaire, including: Have you ever been refused a job you can do/found it difficult to rent an apartment or other residence/refused to enjoy the opportunity to receive education/been excluded from community or social activities/been refused a passport, driver’s license, or other permits due to mental illness in the past 3 months.

#### 2.2.2 Assessment of Physical Aggression Against Others

Physical aggression against others in participants was assessed using the Modified Overt Aggression Scale (MOAS) [24], derived from the widely utilized Overt Aggression Scale (OAS) [25]. The Modified Overt Aggression Scale encompasses four subscales, namely verbal aggression, physical aggression against objects, physical aggression against self, and physical aggression against others. Each subscale comprises four items, with ratings on a 5-point scale (0–4). A score of 0 indicates the absence of corresponding aggressive behavior, while scores ranging from 1 to 4 indicate varying degrees of mild to severe aggressive behavior. In this study, a score of 0 in the physical aggression against others subscale denoted no aggression, and a score greater than 0 indicated aggressive behavior towards others.

#### 2.2.3 Assessment of Childhood Trauma Experiences

This study used a childhood trauma questionnaire to assess potential traumatic events experienced by individuals during childhood. This scale includes 28 items, divided into five dimensions: emotional abuse, physical abuse, sexual abuse, emotional neglect, and physical neglect. Each dimension has five items (a total of 25 items) and the remaining three items are used as validity evaluation items. Each item adopts a 1–5 level scoring system, with each dimension scoring 5–25 points, and the total score range is 25–125 points. The higher the score, the more severe the abuse or neglect suffered by the individual in childhood. If any of the following criteria are met, it is determined to be accompanied by childhood trauma: emotional neglect ≥15 points, emotional abuse ≥13 points, physical abuse >10 points, physical neglect ≥10 points, and sexual abuse ≥8 points [26].

#### 2.2.4 Assessment of Medication Adherence

This study used the Morisky Medication Adherence Scale to assess medication adherence. Its effectiveness and practicality have been validated in patients with schizophrenia, with high internal consistency. The total score is the sum of the scores of the eight questions in the scale, with a score range of 0–8 points. The higher the score, the better the medication adherence: 8 points indicates good compliance; 6–7 points indicates moderate compliance, and <6 points indicates poor compliance [27].

#### 2.2.5 Assessment of Insight and Drug Attitudes

The Insight and Treatment Attitudes Questionnaire aims to measure disease awareness and offer insight into the treatment needs of patients with schizophrenia. It is a single scale consisting of 11 items expressed in the form of questions to elicit answers from the Likert scale, with scores ranging from 0 to 1. The higher the score, the stronger the insight [28].

#### 2.2.6 Assessment of Fatigue Condition

We used a multidimensional fatigue scale to assess the fatigue status of patients with schizophrenia. The scale consists of 20 items and is scored on a scale of 1–5 (with “1” indicating complete non-conformity and “5” indicating complete conformity). It is divided into four subscales: physical fatigue, mental fatigue, decreased motivation, and reduced activity. The higher the total score or factor score, the more severe the individual’s fatigue [13].

#### 2.2.7 Assessment of Discrimination Experience

The Modified Consumer Stigma Questionnaire (MCESQ) developed by Wahl [29] evaluates the discrimination experienced by individuals with schizophrenia due to their mental illness. It consists of 19 items, rated using a 5-point Likert scale (from 1 = rarely to 5 = always), and is divided into two subscales: the Stigma Experience Scale and the Discrimination Experience Scale [29].

### 2.3 Statistical Analyses

The data were statistically analyzed using STATA 15.0 for Windows (Stata Corp, College Station, TX, USA) and R 4.3.2 (R Foundation for Statistical Computing, Vienna, Austria) with packages including “glmnet”, “rms”, and “riskRegression”. For continuous variables, median and interquartile range were used for statistical descriptions, and the Wilcoxon rank-sum test was used for statistical inference. Percentages were used for statistical description of categorical variables, with Fisher’s exact test used as a statistical inference. Variable selection was conducted employing a combination of methods. Specifically, the least absolute shrinkage and selection operator (LASSO) [30], a technique within the realm of machine learning, was utilized. Additionally, multivariate logistic regression analysis was employed for this purpose. While LASSO operates within the domain of machine learning, multivariate logistic regression analysis stands independently in its traditional statistical framework. This hybrid approach allowed for a comprehensive examination of the variables, leveraging the strengths of both methodologies to form two predictive models. Specifically, for the LASSO method, solutions were derived through 10-fold cross-validation. LASSO estimates were obtained using both the lasso(min) and the lasso(1SE) criteria [31]. Additionally, numerous multivariate logistic regression analyses were conducted. Given the study’s applicability, a simple and convenient model was required, leading to the selection of the Bayesian information criterion (BIC) as the stopping rule [32].

To assess and compare the performance of models on external data, three metrics were calculated for models generated from two different variable selection approaches. In the testing dataset, the area under the receiver operating characteristic (ROC) curve was computed to quantify discriminatory performance. Calibration was performed using the Unreliability test was conducted using R 4.3.2 with the rms package. Decision curve analysis was conducted to determine the clinical utility of the models, quantifying the net benefit at different threshold probabilities in the testing dataset [33]. Finally, a nomogram based on a simple and convenient predictive model was developed to facilitate visualization for use by mental health nursing professionals in rural communities. A *p*-value less than 0.05 on both sides was considered statistically significant.

## 3. Results

### 3.1 Participant Characteristics

**Fig. 1.**
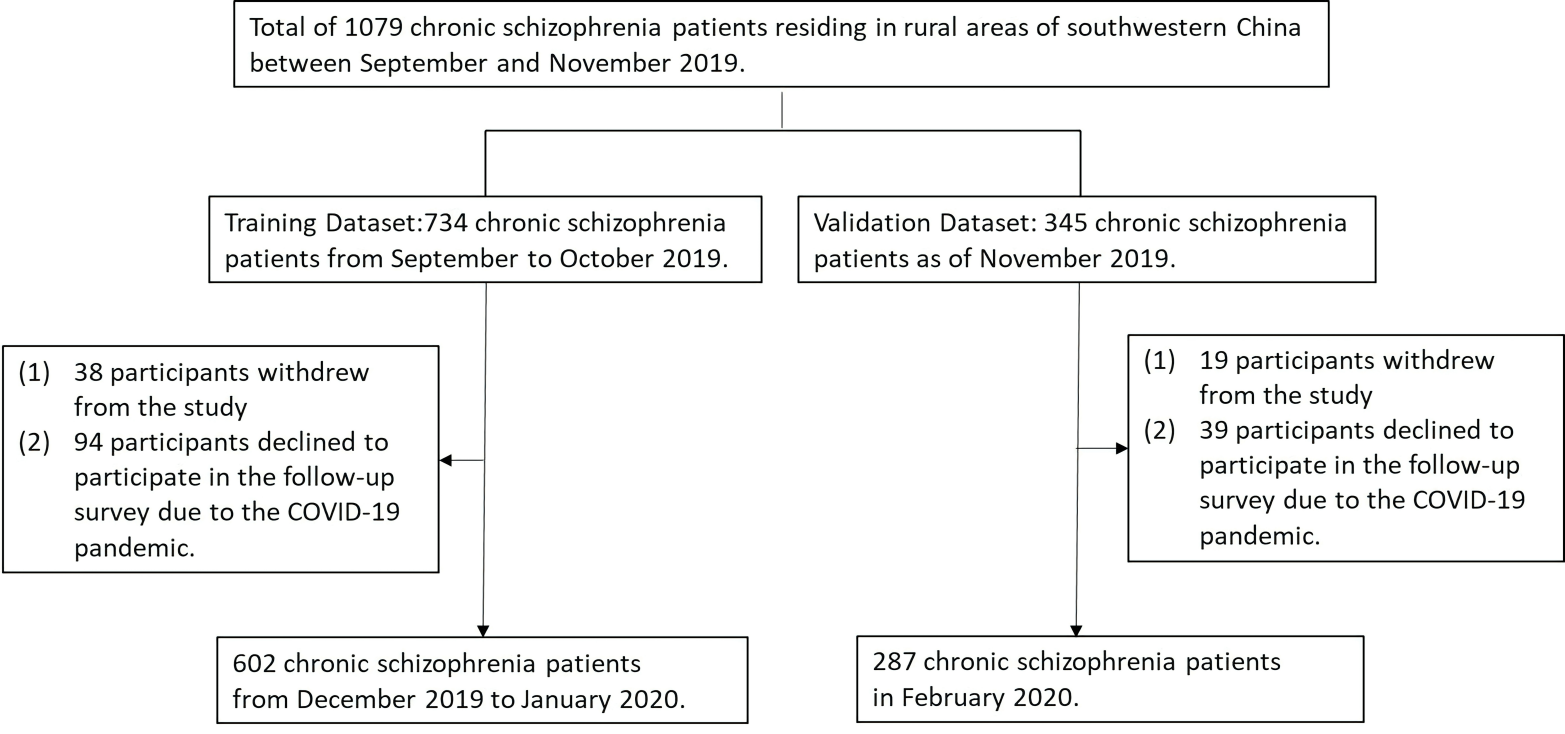
**Flowchart outlining the recruitment procedure**.

A total of 1079 participants meeting the study criteria participated in the baseline survey, with 889 (82.39%) completing the follow-up survey. Among the 190 participants lost to follow-up, 57 refused to participate and 133 refused to participate in the investigation because of the COVID-19 pandemic (see Fig. 1). Among the 889 participants, 477 were male and a total of 258 individuals (29.02%) reported engaging in physical aggression against others in the past 3 months. For the demographic and disease-related information of the participants in the training and testing datasets, refer to Table 2.

**Table 2.** **Characteristics of the participants in the training and testing datasets (N = 889)**.

Characteristic		Training Dataset		*p*	Testing Dataset		*p*
		Physical aggression against others, no (n = 422)	Physical aggression against others, yes (n = 180)		Physical aggression against others, no (n = 209)	Physical aggression against others, yes (n = 78)	
		n (%)	n (%)		n (%)	n (%)	
Gender				0.017			0.097
	Male	192 (45.5)	101 (56.1)		140 (67.0)	44 (56.4)	
	Female	230 (54.5)	79 (43.9)		69 (33.0)	34 (43.6)	
Birthplace				0.157			0.877
	Rural	374 (88.6)	152 (84.4)		181 (86.6)	67 (85.9)	
	Urban	48 (11.4)	28 (15.6)		28 (13.4)	11 (14.1)	
Living alone				0.230			<0.001
	No	388 (91.9)	160 (88.9)		170 (81.3)	76 (97.4)	
	Yes	34 (8.1)	20 (11.1)		39 (18.7)	2 (2.6)	
Marital status				0.005			0.313
	No	175 (41.5)	97 (53.9)		162 (77.5)	56 (71.8)	
	Yes	247 (58.5)	83 (46.1)		47 (22.5)	22 (28.2)	
Employment				0.184			0.396
	No	374 (88.6)	166 (92.2)		200 (95.7)	72 (92.3)	
	Yes	48 (11.4)	14 (7.8)		9 (4.3)	6 (7.7)	
Household monthly income (USD)				0.158			0.055
	≤689.59	393 (93.1)	173 (96.1)		178 (85.2)	73 (93.6)	
	>689.59	29 (6.9)	7 (3.9)		31 (14.8)	5 (6.4)	
Smoking				0.031			0.739
	No	111 (26.3)	63 (35.0)		68 (32.5)	27 (34.6)	
	Yes	311 (73.7)	117 (65.0)		141 (67.5)	51 (65.4)	
Alcohol abuse				0.444			1.000
	No	375 (88.9)	156 (86.7)		197 (94.3)	74 (94.9)	
	Yes	47 (11.1)	24 (13.3)		12 (5.7)	4 (5.1)	
Substance abuse				0.739			0.886
	No	420 (99.5)	178 (98.9)		206 (98.6)	76 (97.4)	
	Yes	2 (0.5)	2 (1.1)		3 (1.4)	2 (2.6)	
Perceived family economic status				0.048			0.140
	Very bad	26 (6.2)	20 (11.1)		23 (11.0)	6 (7.7)	
	Bad	124 (29.4)	63 (35.0)		68 (32.5)	23 (29.5)	
	Neutral	238 (56.4)	87 (48.3)		100 (47.8)	47 (60.3)	
	Good	34 (8.1)	10 (5.6)		18 (8.7)	2 (2.6)	
Perceived social status				0.773			0.914
	Very low	24 (5.7)	13 (7.2)		24 (11.5)	7 (9.0)	
	Low	95 (22.5)	45 (25.0)		63 (30.1)	24 (30.8)	
	Neutral	290 (68.7)	117 (65.0)		115 (55.0)	45 (57.7)	
	High	13 (3.1)	5 (2.8)		7 (3.4)	2 (2.6)	
First episode schizophrenia				0.244			<0.001
	No	272 (64.5)	107 (59.4)		155 (74.2)	37 (47.4)	
	Yes	150 (35.5)	73 (40.6)		54 (25.8)	41 (52.6)	
Age* (years), Median (P25, P75)		47.00 (38.00, 52.00)	46.00 (35.00, 51.00)	0.240	48.00 (42.00, 53.00)	44.50 (31.00, 50.00)	0.002
Years of education*, Median (P25, P75)		9.00 (6.00, 9.00)	8.00 (6.00, 9.00)	0.480	6.00 (6.00, 9.00)	7.50 (6.00, 9.00)	0.190
Body mass index*, Median (P25, P75)		24.39 (21.72, 27.01)	24.53 (21.48, 27.29)	0.820	24.03 (21.48, 26.42)	24.07 (21.90, 26.60)	0.200
Number of antipsychotic medications taken in the past 3 months*, Median (P25, P75)		1.00 (1.00, 1.00)	1.00 (1.00, 2.00)	0.110	1.00 (1.00, 1.00)	1.00 (1.00, 1.00)	0.920
Schizophrenia duration, (monthly)*, Median (P25, P75)		156.50 (106.00, 252.00)	165.50 (96.50, 263.50)	0.930	161.00 (83.00, 253.00)	144.00 (84.00, 246.00)	0.570

*Calculated using the Wilcoxon rank-sum test.

### 3.2 Prediction Model Development

**Fig. 2.**
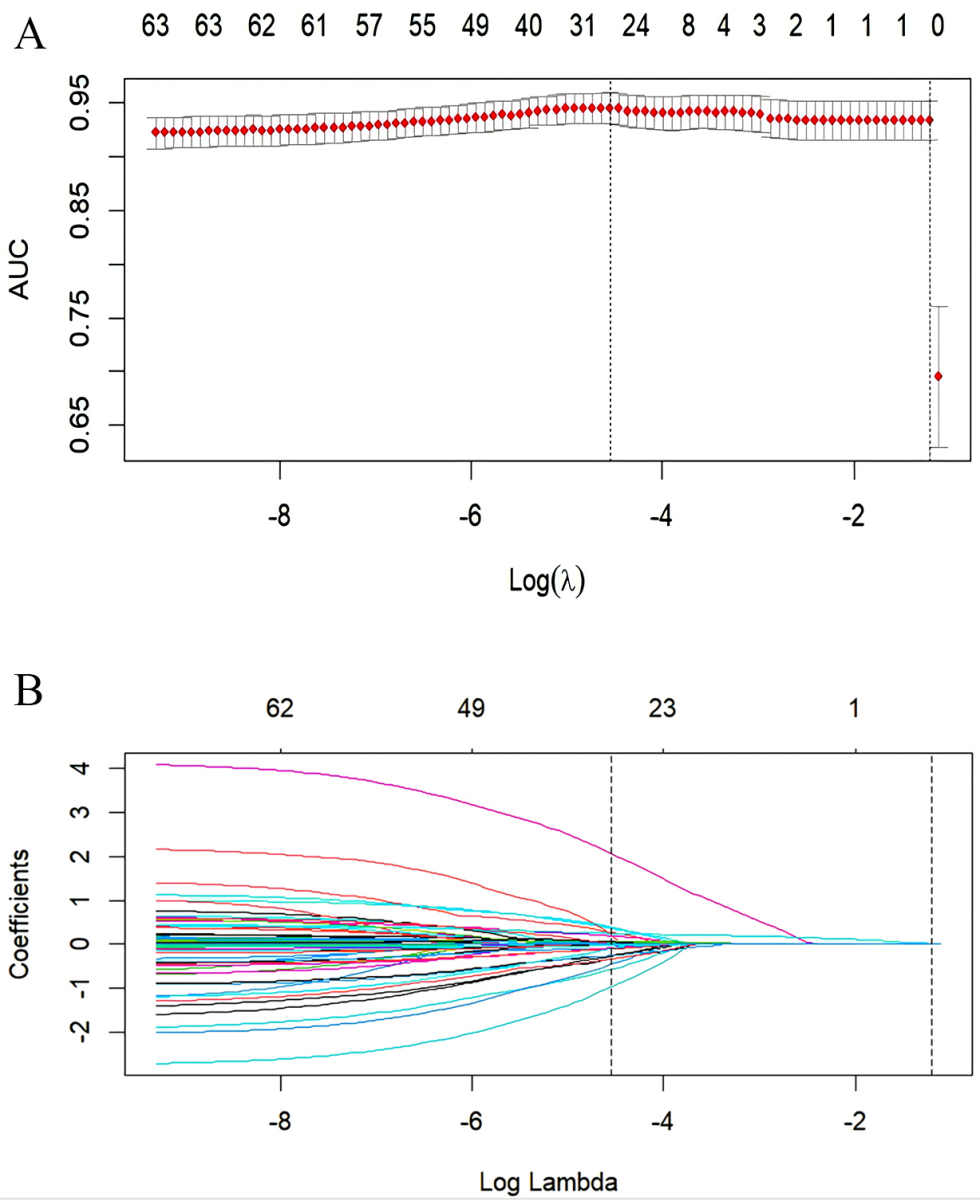
**Variable selection using the least absolute shrinkage and selection operator (LASSO) on the training dataset**. (A) In the LASSO model, the tuning parameter (λ) selection was conducted using 10-fold cross-validation based on minimum criteria. The area under the receiver operating characteristic (AUC) curve was plotted against the logarithm of (λ). Dotted vertical lines indicate the optimal values determined by both the minimum criteria and the 1 standard error of the minimum criteria (1-SE criteria). A value of 0.01057 for λ was chosen based on the minimum criteria determined through 10-fold cross-validation. (B) A coefficient profile plot was generated for the 62 features using the LASSO method. The plot displays the coefficients against the log (λ) sequence. A vertical line indicates the value chosen through 10-fold cross-validation, where the minimum value of λ led to 27 nonzero coefficients and the 1-SE value of λ led to one nonzero coefficient.

Fig. 2 illustrates that when using the LASSO algorithm for variable selection, 27 variables were selected from a pool of 62 candidate factors to forecast physical aggression against others when lambda was at its minimum (Model 1) (λ = 0.01057). These 27 variables are detailed in Table 3. However, when lambda was set at one standard deviation, only one variable was selected, namely the MOAS score at the first episode of schizophrenia. Consequently, subsequent performance exploration and comparisons were conducted exclusively on the predictive model with the 27 variables.

**Table 3.** **Risk factors for physical aggression against others in patients with schizophrenia in rural communities**.

	Model 1			Model 2		
Intercept and Variable	β	Odds Ratio (95% CI)	*p*	β	Odds Ratio (95% CI)	*p*
Intercept	–4.089		0.188	–9.561		<0.001
FESPAAOS^a^	3.957	52.319 (9.080, 301.446)	<0.001	3.396	29.845 (7.288, 122.226)	<0.001
FESMOAS^a^	0.376	1.457 (1.356, 1.565)	<0.001	0.295	1.343 (1.276, 1.413)	<0.001
MF	0.073	1.075 (0.954, 1.213)	0.236	0.127	1.135 (1.032, 1.248)	0.009
BMI	0.172	1.188 (1.089, 1.297)	<0.001	0.097	1.102 (1.028, 1.180)	0.006
DS	0.085	1.089 (1.030, 1.151)	0.003	0.079	1.082 (1.034, 1.132)	0.001
FESAAO^a^	–1.550	0.212 (0.055, 0.816)	0.024	–1.956	0.141 (0.044, 0.457)	0.001
Gender	–0.664	0.515 (0.236, 1.125)	0.096	NA	NA	NA
Birthplace	0.846	2.330 (0.824, 6.590)	0.111	NA	NA	NA
Living with parents*	–1.349	0.259 (0.068, 0.987)	0.048	NA	NA	NA
Living alone	0.598	1.819 (0.572, 5.787)	0.311	NA	NA	NA
Employment	–0.850	0.428 (0.125, 1.457)	0.174	NA	NA	NA
Having one or more sexual partners	–0.233	0.792 (0.363, 1.728)	0.558	NA	NA	NA
First episode of schizophrenia	1.056	2.876 (1.390, 5.950)	0.004	NA	NA	NA
FESAAS^a^	–2.660	0.070 (0.012, 0.415)	0.003	NA	NA	NA
Substance abuse	1.987	7.292 (0.240, 221.645)	0.254	NA	NA	NA
Mother with schizophrenia	0.956	2.600 (0.984, 6.869)	0.054	NA	NA	NA
Mother smoked *	–0.963	0.382 (0.096, 1.518)	0.172	NA	NA	NA
Father drank alcohol *	–0.903	0.405 (0.167, 0.983)	0.046	NA	NA	NA
Years of education	–0.063	0.939 (0.848, 1.039)	0.221	NA	NA	NA
Perceived family economic status	–0.477	0.621 (0.389, 0.991)	0.046	NA	NA	NA
Parental relationship *	–0.421	0.656 (0.412, 1.045)	0.076	NA	NA	NA
Childhood emotional neglect ^b^	–0.064	0.938 (0.864, 1.019)	0.130	NA	NA	NA
Attitude towards medication ^c^	–0.327	0.721 (0.557, 0.933)	0.013	NA	NA	NA
Insight and treatment attitudes ^d^	0.080	1.083 (1.026, 1.143)	0.004	NA	NA	NA
Anxiety factor in the BPRS	–0.767	0.464 (0.261, 0.826)	0.009	NA	NA	NA
Psychosis factor in the BPRS	0.142	1.153 (0.647, 2.055)	0.630	NA	NA	NA
Activation factor in the BPRS	0.506	1.658 (0.839, 3.278)	0.146	NA	NA	NA

Note: β is the regression coefficient. * Refers to the situation before the age of 16 years in patients with schizophrenia. ^a^ Measured by the Modified Overt Aggression Scale. ^b^ Measured by the Childhood Trauma Questionnaire. ^c^ Measured by the Medication Adherence Rating Scale. ^d^ Measured by the Insight and Treatment Attitudes Questionnaire. Abbreviations: FESPAAOS, physical aggression against others before first episode of schizophrenia; FESMOAS, scores on the Modified Overt Aggression Scale at the onset of the first episode of schizophrenia; MF, mental fatigue score of the Multidimensional Fatigue Inventory-20; BMI, body mass index; DS, discrimination experience score of the Modified Consumer Experiences of Stigma Questionnaire; FESAAO, aggression against objects before the first episode of schizophrenia; FESAAS, aggression against self before the first episode of schizophrenia; BPRS, Brief Psychiatric Rating Scale; NA, not applicable.

Through multivariate stepwise logistic regression analysis, six predictors were identified (Model 2). These predictors included body mass index, incidents of aggression against objects prior to the first episode of schizophrenia, incidents of aggression against others prior to the first episode of schizophrenia, the score on the MOAS at the onset of the first episode of schizophrenia, experiences of discrimination, and mental fatigue (as detailed in Table 3).

### 3.3 Prediction Model External Validation

**Fig. 3.**
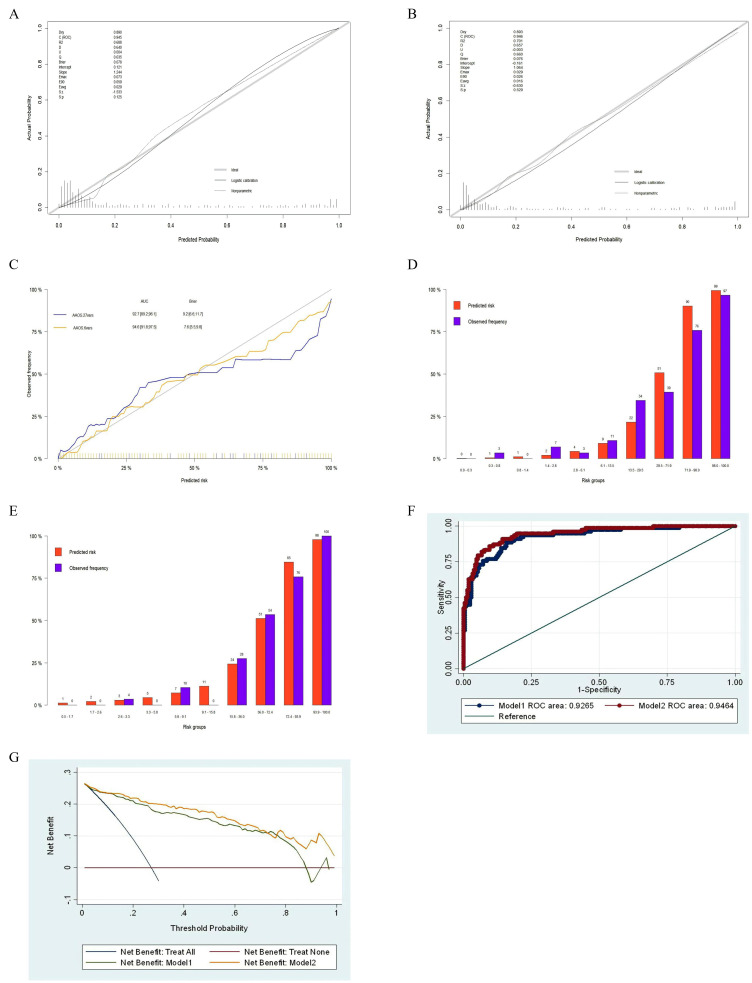
**Efficacy of the prediction models for the risk of physical aggression against others among schizophrenia patients in rural communities in the test dataset**. (A) Calibration plot of Model 1. (B) Calibration plot of Model 2. (C) Comparison of the predictive accuracy between Model 1 and Model 2. (D) Frequency plot of the calibration of Model 1. The x-axis represents the predicted risk, divided into 10 risk bins (q = 10) according to magnitude, while the y-axis represents the observed frequency. The risk bins range from 0.3% to 0.8% for the second bin, with a predicted probability of 1% and an actual frequency of 3%; for the tenth bin, the risk ranges from 98.0% to 100.0%, with a predicted probability of 99% and an actual frequency of 97%. (E) Frequency plot of the calibration of Model 2. The x-axis represents the predicted risk, divided into 10 risk bins (q = 10) according to magnitude, while the y-axis represents the observed frequency. The risk bins range from 2.6% to 3.3% for the third bin, with a predicted probability of 3% and an actual frequency of 4%; for the tenth bin, the risk ranges from 93.9% to 100.0%, with a predicted probability of 98% and an actual frequency of 100%. (F) Receiver operating characteristic (ROC) analysis of Model 1 and Model 2 area under the curve (AUC). (G) Decision curve analysis for Model 1 and Model 2.

**Fig. 4.**
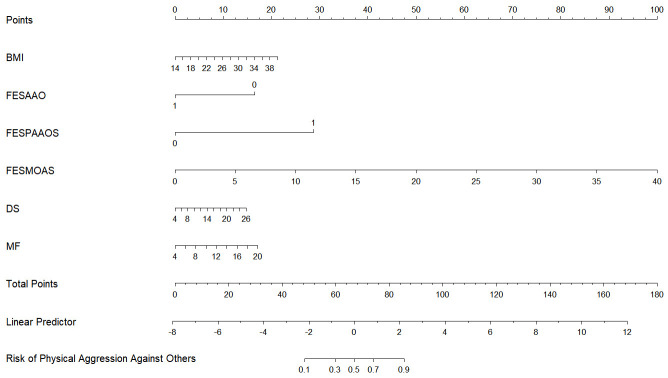
**Nomogram for Model 2**. Note: FESAAO (1 = Yes, 0 = No); FESPAAOS (1 = Yes, 0 = No).

In the prediction models with 287 participants in the testing dataset, Fig. 3C,F show that the area under the ROC curve was 0.927 (95% CI, 0.892–0.961) for Model 1 and 0.946 (95% CI, 0.918–0.975) for Model 2, indicating perfect discrimination in both models. In evaluating calibration, Fig. 3A,B show that Model 1’s Brier score was 0.078, Emax was 0.073, E90 was 0.059, and Eavg was 0.029, with a *p*-value of 0.125; while Model 2’s Brier score was 0.076, Emax was 0.029, E90 was 0.026, and Eavg was 0.016, with a *p*-value of 0.529. This indicates that both models passed the Unreliability test. Additionally, as evident from Fig. 3A–C, and the frequency plots of calibration Fig. 3D,E, both models exhibit good predictive accuracy. In addition, the decision curve analysis (DCA) indicates that when the threshold probability of risk of aggression against others ranged from 1% to 88%, Model 1 provided more benefits than ‘None’ or ‘All’. Similarly, for Model 2, the net benefit exceeded ‘None’ or ‘All’ when the threshold probability ranged from 1% to 100% (see Fig. 3G). Moreover, The Akaike Information Criterion (AIC) was 340.118, and the Bayesian Information Criterion (BIC) was 370.919 in Model 2, and a nomogram was developed to visually represent these six predictive factors for physical aggression against others in schizophrenia (refer to Fig. 4).

## 4. Discussion

This study developed two prediction models using a LASSO algorithm and multivariate logistic regression analysis respectively to predict the risk of physical aggression against others among schizophrenia patients in rural communities in southwestern China within a 3-month period. Through model comparison, the six-variable model was found to offer greater convenience for community risk screening without compromising model performance. These six predictive variables include three historical factors and three dynamic, modifiable factors.

The historical record of aggressive behavior associated with schizophrenia has consistently served as a stable and significant predictor. This study found that individuals with high MOAS scores at the onset of the first episode and those with a history of prior physical aggression towards others before the first episode of schizophrenia displayed significant predictive capacity for future physical aggression. A study conducted in China revealed that 40% of patients with first-episode psychosis who exhibited physical aggression towards others before treatment continued to engage in this way for 3 years after treatment [34]. Nevertheless, this study showed an unexpected outcome—rural community schizophrenia patients who had engaged in aggression against objects before the first onset of schizophrenia was, in fact, shown to be a protective factor mitigating the risk of subsequent aggressive behaviors towards others. This phenomenon could be attributed to the selection of community-based samples in this study. Individuals diagnosed with schizophrenia who persist in exhibiting violent behavior towards others are more prone to being hospitalized for treatment rather than in the community. Individuals diagnosed with schizophrenia in this study were distinguished by a median duration surpassing 10 years. In addition, the majority lived with their families and possessed a cognitive ability that was generally adequate to respond to questionnaires. Personality consistently plays a pivotal role in the adoption of coping strategies [35], indicating that individuals tend to manifest adaptive behaviors in response to stressful situations in a consistent and stable manner. Regarding coping strategies, individuals with schizophrenia may not exhibit as pronounced differences as one might anticipate when compared with their healthy counterparts [36]. Schizophrenic patients residing in the community might lean towards employing coping strategies involving property damage rather than engaging in ‘aggression against others’. This preference might arise from the awareness that the latter was more likely to result in restrictions or hospitalization.

Three dynamic factors of notable concern were identified for predicting the risk of physical aggression against others among rural community schizophrenia patients in this study. Body mass index (BMI) was an important predictor, which is consistent with previous findings [37]. Weight gain accelerated upon initiation of antipsychotic treatment and persisted thereafter for patients with severe mental illness [38]. The association between BMI and aggression might be connected to abnormal lipid metabolism and plasma inflammatory cytokine levels [39, 40, 41]. Furthermore, an increased BMI could potentially disrupt white matter in the brains of individuals with schizophrenia, hampering the structural connectivity of crucial cortico-limbic networks and consequently impacting neurocognitive function and emotional processing abilities [42]. Obesity might also be linked to aggressive behavior through heightened concerns and anxiety regarding body size. It is worth mentioning that the relationship between obesity and violence exists not only among schizophrenia patients but also in the general population [43], including adolescents [44]. Nevertheless, individuals with severe mental illness demonstrate a notably higher prevalence and greater likelihood of obesity than the general population [45]. In the Asia-Pacific region, metabolic syndrome is twice as prevalent among schizophrenia patients living in the community compared with the general population [46].

Importantly, this study examined five manifestations of discrimination related to mental illness, encompassing employment, home rental, access to education, participation in social activities, and application for drivers’ licenses. In addition, the elevated frequency of discrimination experiences within the past 3 months correlated with an increased risk of engaging in aggressive behavior towards others. Rates of anticipated and experienced discrimination remain consistently high among individuals with mental illness across various countries [47, 48]. Individuals diagnosed with schizophrenia who live in rural areas in developing countries often experience more instances of ridicule, offensive comments, rejection, and differential treatment by society [49]. Experiencing discrimination is also the most significant contributing factor to internalized stigma [50]. Due to the potential impairment of attention, memory, and other functions in patients with schizophrenia, some predictive factors in this study relied on retrospective self-reported data, which can lead to recall bias [51]. Despite limited research on the relationship and mechanisms between experiencing discrimination and increased aggression, their potential influence appears to be linked to the elicitation of anger [52].

This study also found that mental fatigue was a predictor of physical aggression against others among schizophrenia patients residing in rural communities. The Multidimensional Fatigue Inventory-20 was employed in this study to assess the fatigue of schizophrenia patients residing in rural communities, a scale widely utilized to evaluate mental fatigue in individuals with psychiatric disorders [53]. This mental fatigue factor was primarily characterized by difficulties in concentration or being easily distracted. Mental fatigue is a psychobiological condition associated with impaired cognitive and physical performance across various settings [54]. Previous studies have shown that decreased cognitive abilities, including attention, are associated with an increased likelihood of aggressive behavior in schizophrenic inpatients [55] and in mentally disordered offenders [56]. Notably, mental fatigue predicts aggression not only among schizophrenia patients but also in other populations; a previous study showed that mental healthcare professionals’ mental fatigue significantly predicts their aggressive behavior [57]. The follow-up period of this study also coincided with the outbreak of the COVID-19 pandemic. Relevant studies have shown a significant correlation between the onset of psychiatric symptoms and exposure to the coronavirus [58]. The analysis suggests that this may be due to the psychological burden that schizophrenia patients may have faced during the pandemic, making them more prone to anxiety and aggressive behavior [59].

Additionally, model comparisons revealed that the 6-variable model offers greater convenience for community risk screening without compromising model performance. This may be attributed to some recognized limitations of LASSO regularization that could restrict its applicability to psychological research [60].

### Limitations

The participants in this study were schizophrenia patients residing in rural communities in southwestern China. While the predictive models developed in this study hold practical significance for the community management and follow-up of schizophrenia patients in China, a densely populated agricultural country, their applicability to diverse cultures and ethnicities worldwide is evidently limited. The model used in this study relies only on the training and validation sets, which may increase the risk of overfitting. In future research, we will expand the scope of objects in the training and validation sets to reduce the risk of overfitting. Further, this study incorporated 62 factors as candidate contributors to aggression against others in schizophrenia, yet there were still additional factors that were not taken into consideration. In addition, the retrospective data in the questionnaires might be subject to recall bias. Another limitation of this study was the lack of documentation of patients’ aggression status at baseline assessment. This was due to the practical reality in community schizophrenia follow-up management, where patients were typically initially included in mental health nursing professional community management without prior assessment of aggression status. However, this still resulted in the inability of the models developed in this study to assess the impact of aggression status 3 months prior on the current aggression status. Furthermore, the convenience sampling method used in this study raises concerns about sample representativeness.

## 5. Conclusion

The prediction models developed and validated in this study for the risk of aggression against others in patients with schizophrenia exhibited good predictive performance. Compared to the model with 27 variables, the model with six variables demonstrated better practical utility for community management. These six predictive variables include three historical factors and three dynamic, modifiable factors. It is worth noting that, apart from the MOAS score at the onset of the first episode of schizophrenia, the other predictive factors appeared to be non-specific to schizophrenia. These findings may help to alleviate social discrimination and constraints faced by individuals with schizophrenia in rural communities of southwestern China, facilitating the provision of proactive mental health services. Furthermore, it underscores that body mass index, experiences of discrimination, and mental fatigue emerged as critical areas for community mental health nursing professionals to address.

## Availability of Data and Materials

The data that support the findings of this study are available from the corresponding author upon reasonable request.
